# Urinary Ultrasound and Other Imaging for Ureteropelvic Junction Type Hydronephrosis (UPJHN)

**DOI:** 10.3389/fped.2020.00546

**Published:** 2020-09-16

**Authors:** Ayse Kalyoncu Ucar, Sebuh Kurugoglu

**Affiliations:** ^1^Istanbul Kanuni Sultan Suleyman Training and Research Hospital, Istanbul, Turkey; ^2^Istanbul University Cerrahpasa Faculty of Medicine, Istanbul, Turkey

**Keywords:** child, UPJ type hydronephrosis, ultrasonography, CT angiography, MR urography

## Abstract

Ultrasound is the main imaging study used to diagnose ureteropelvic junction (UPJ) obstruction. On ultrasound, abnormal dilatation of the pelvicalyceal system of varying degrees is seen, whereas the ureter is normal in caliber. A properly performed study provides essential information regarding laterality, renal size, thickness, and architecture of the renal cortex and degree of dilatation of the pelvicalyceal system. Doppler ultrasound may identify a crossing vessel, when present. This imaging method also has been used differentiating obstructive from non-obstructive hydronephrosis by renal arterial resistive index measurements. Abdominal radiographs may show soft tissue fullness, bulging of the flank, and displacement of bowel loops from the affected side. The voiding/micturating cystourethrogram helps exclude other causes of upper tract dilatation, including vesicoureteral reflux, urethral valves, and ureteroceles. Computerized Tomography angiography with multiplanar reformation and three-dimensional images may be used to depict suspected crossing vessels as a cause of UPJ obstruction in older children and adults. Magnetic Resonance Urography has progressed significantly in recent years due to the development of both hardware and software that are used to generate high-resolution images. This imaging technique currently allows for the detailed assessment of urinary tract anatomy, while also providing information regarding renal function, including differential renal function, and the presence or absence of obstructive uropathy.

## Introduction

Ureteropelvic junction (UPJ) obstruction is the most common cause of pathologic obstructive hydronephrosis in children which is defined as a partial or complete obstruction of the flow of urine from the renal pelvis to the proximal ureter (1, 2). Many theories have been put forward to explain the pathophysiology; however, the cause is not clear. As an intrinsic cause of obstruction abnormally developed ureteral smooth muscle at the UPJ resulting in an aperistaltic segment is considered, while extrinsic obstruction is thought to be caused by an overlying renal vessel (3, 4). UPJ obstruction might lead to progressive damage to the renal function by increasing back pressure on the kidney (5). But the majority of cases resolve spontaneously without a real obstruction and renal damage. Especially in newborns and infants, hydronephrosis develops as a useful adaptation mechanism that protects the kidney from high pressure and damage secondary to the good compliance of the renal pelvis, not as a result of obstruction (6). Therefore, the differentiation of true obstruction from urinary tract dilatation is crucial to avoid unnecessary surgical intervention. All efforts are made to recognize which cases to follow and which ones to treat. Imaging methods play an important and crucial role at this point.

The purpose of this review is to discuss the radiological findings of hydronephrosis related to UPJ obstruction under the title of “ureteropelvic junction type hydronephrosis (UPJHN),” based mainly on ultrasonography and other imaging methods.

## Ultrasonography

Ultrasonography (US) is the main imaging study used for evaluating the urinary system in the postnatal period in children with suspected or diagnosed prenatal hydronephrosis (7). This method has lots of advantages such as being safe and non-invasive, cheap, easily accessible in most institutions and also being repeatable with using no radiation exposure. The widespread use of antenatal US screening leads to a significant increase in the detection rate of UPJHN (8). All newborns with a history of antenatal hydronephrosis should be evaluated by US in postnatal period (9). If US is performed in the first postnatal days, mild hydronephrosis may not be detected or the degree of hydronephrosis may appear milder than the fact due to transient dehydration as a result of physiological oliguria in the early postnatal period and subsequent polyuria. Therefore, it is more appropriate to perform the first urinary US examination usually after first week of birth (10, 11). However, in cases of bilateral hydronephrosis, severe hydronephrosis in a solitary kidney, elevated creatinine levels, urinary tract infection, suspected perforation, or posterior urethral valve, early neonatal US may require urgency. If postnatal US is normal, it should be repeated after 4–6 weeks (9). For instance, data in a study shows that 5% of patients requiring surgery for obstructive uropathies had abnormal US findings at 1 month of age despite normal US findings at 1 week of age (12).

A variety of (multifrequency) transducers are used in the evaluation of pediatric urinary tract. For standard pediatric exams, both use of convex probes ranging from 2.5 to 10 MHz and linear probes ranging from 5 to 17 MHz are advisable. High-frequency, high-resolution linear probes are necessary for evaluating details or for assessing neonatal patients. Each kidney should be assessed both in transverse and longitudinal planes. In addition to supine and decubitus positions, prone position reduces the distance to the kidneys, increases image quality, provides better image quality, and enables the medullary structure to be better evaluated (13).

In the presence of UPJHN, US demonstrates multiple dilated calyces of uniform size which communicate with a dilated renal pelvis and abrupt narrowing at the level of the UPJ whereas the ureter is normal in caliber (14). Dilatation may vary depending on position, hydration, fullness of bladder, and kidney function. In the setting of dilatation, the patient should be reexamined after emptying bladder in order to assess the exact severity of dilatation. Since the position of the patient is one of the factors affecting hydronephrosis evaluation, the same position should be used for each follow-up measurement to make accurate comparisons (15).

In addition to ensuring an accurate determination of hydronephrosis, sonographic evaluation has an important role in determining the timing and necessity of other examinations. Since most unnecessary nuclear imaging and voiding cystourethrography examinations are mainly caused by inadequate or inaccurrate information in US reports, a detailed and well-performed US can minimize unnecessary invasive tests that seriously concern children and their parents.

**Figure 1:**
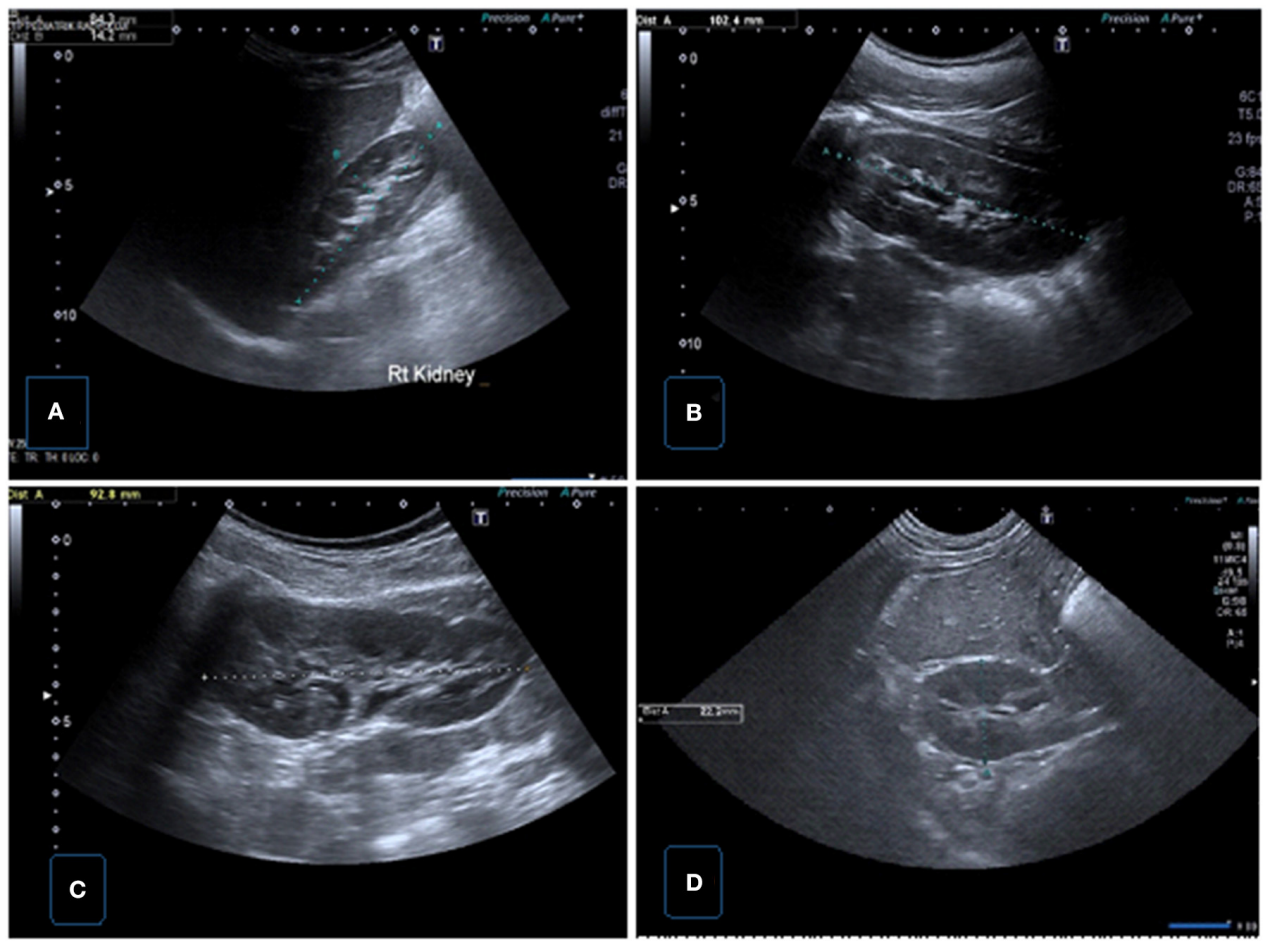
Normal renal sonographic images obtained with convex probes. **(A)** Longitudinal US image of the right kidney demonstrating renal length and parenchymal thickness in supine position. **(B)** Longitudinal US image of the left kidney demonstrating renal length in supine position. **(C)** Longitudinal US image of the left kidney demonstrating renal length in prone position. **(D)** Transverse US image of the right kidney showing renal AP size.

**Figure 2:**
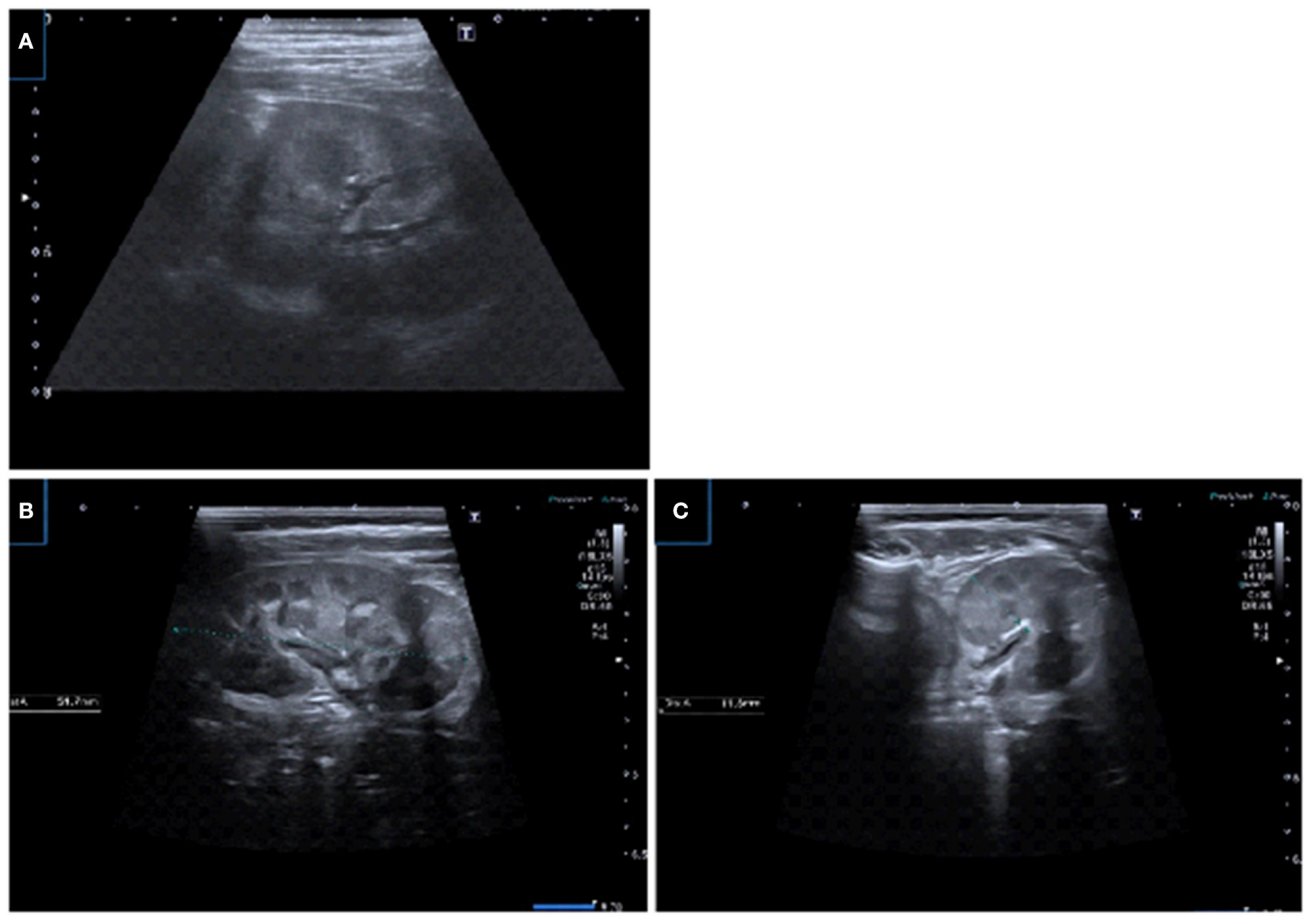
US images using linear transducers. **(A)** Transverse US image demonstrating corticomedullary differentiation in prone position and detailed visualization of the parenchyma. **(B,C)** Renal longitudinal and transverse US images in prone position demonstrating physiologic medullary echogeneity with corticomedullary differentiation and uroepithelial thickening in pelvis.

US examination provides essential information regarding laterality, kidney size, appearance (such as echogenicity, corticomedullary differentiation, cyst), parenchymal thickness, presence of pelvicalyceal dilatation (Figure 1) (7, 13, 16, 17). High frequency linear transducers maximize the sonographic resolution of the kidney enabling better evaluation of the medulla and cortex (Figure 2) (13). US also gives important information about contralateral kidney, ureter, and bladder. Due to the increased incidence of other congenital abnormalities of the urinary tract in patients with UPJ obstruction such as vesicoureteral reflux, renal duplication, ureterovesical obstruction, and bilateral UPJ obstruction (10%) (5, 18), a properly performed study should include all the necessary data. However, this is directly correlated to the practitioners training and experience.

US examination is important to determine the exact level and severity of obstruction in patients with UPJHN, the appropriate treatment, and follow-up decision. This imaging method should be performed periodically at varying intervals according to the severity of hydronephrosis. The primary aim of treatment is to prevent or minimize renal damage and loss of function. In order to ensure the right decision regarding the necessity of surgery and follow- up, some measurements and grading systems have been developed (19–22). The most commonly approved sonographic measurement systems to assess hydronephrosis are the anterio-posterior renal pelvic diameter (APRPD), the Society for Fetal Urology (SFU) grading system, the Urinary Tract Dilation (UTD) system, and the Onen classification.

## Anterio-Posterior Renal Pelvic Diameter

**Figure 3:**
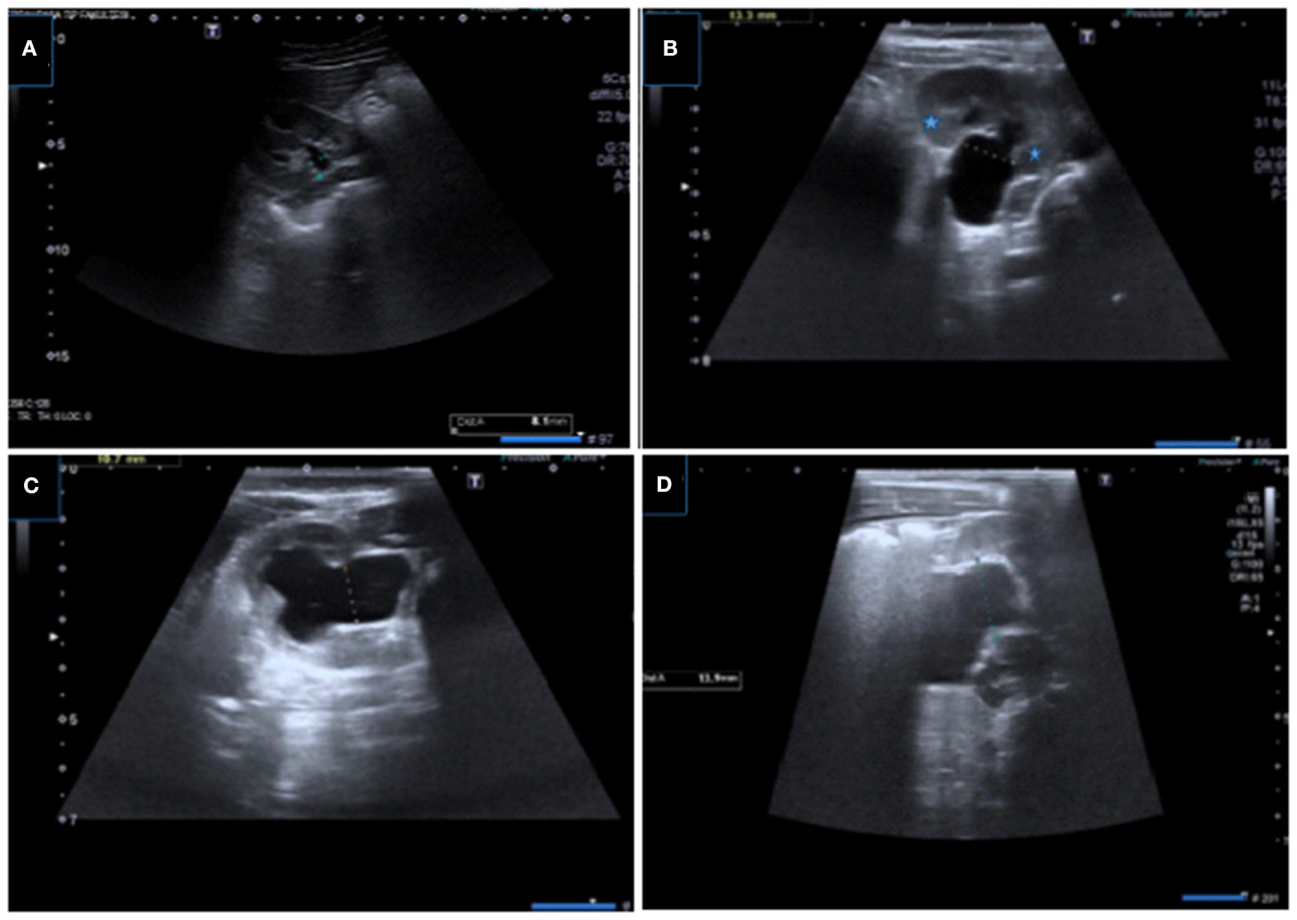
Renal US images showing measurement of APRPD with different grades of hydronephrosis. **(A–D)** Samples of optimal APRPD measurements obtained within the confines of the renal cortex in transverse plane.

**Figure 4:**
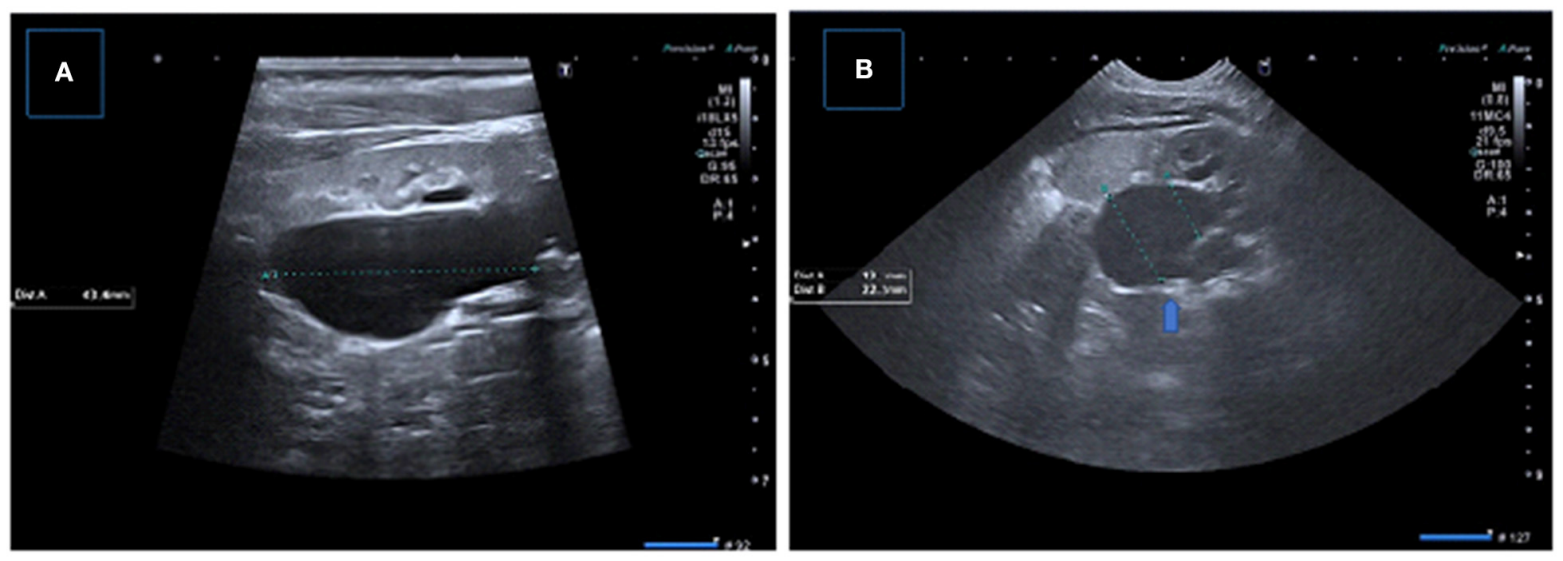
Incorrect measurement of APRPD in longitudinal **(A)** and transverse **(B)** US images showing incorrect measurement at extrarenal level (arrow), correct measurement level is also shown.

Anterio-posterior renal pelvic diameter (APRPD) is a quantitative parameter based on the measurement of the greatest diameter on US images acquired in a transverse plane in order to assess the degree of dilatation of the renal pelvis (Figure 3) (22, 23). Monitoring the degree of pelvic dilatation is an important aspect of follow-up in UPJHN. Measurement of APRPD is commonly used as a comparable and sensitive parameter. But this measurement is not fully standardized among radiologists. The most common mistake is to measure the pelvis in longitudinal plane or from the widest extrarenal level (Figure 4). Even if the APRPD measurement is performed optimally, it may vary depending on the hydration status, the bladder being full/empty and the position where it is measured (supine or prone). Hydration can increase renal pelvic dilatation by causing fluctuation in bladder volume and an increase in fluid excretion (24, 25). Although there is no standard renal sonogram protocol regarding hydration status in the evaluation of pediatric hydronephrosis, the effect of hydration on the diameter of the pelvis has been well-documented (25). Hasch (26) recommended a fasting US scan in order to exclude a persistent hydronephrosis, as well as a reassessment after fluid intake so as not to overlook a case of intermittent hydronephrosis. However, performing this method on infants and younger children is not a simple task.

The accurate measurement of APRPD can be affected by patient position. According to Sharma et. al's study in many cases the APRPD decreases when measured in the prone position (15). US done in the prone and supine positions can also help to differentiate non-obstructive dilatation from obstructive dilatation. While a non-obstructive dilated pelvis can drain better in the prone position, obstructive systems cannot. The measurement of APRPD in the supine and prone positions does not change in the setting of obstruction (15).

Besides the disadvantage of the dynamic nature of APRPD, it is not sufficient alone as it does not provide information about the presence of abnormal renal morphology, parenchymal integrity, or tension in the calices (27).

In some cases, there may be a serious difference between the measurement of APRPD and the actual degree of hydronephrosis, deeming it essential to indicate whether the pelvis is extrarenal or intrarenal, as the kidneys with extrarenal pelvis have lower parenchymal damage by keeping the pressure low for longer. If APRPD is measured from the extrarenal level, it may be perceived as having a more severe obstruction than in actuality (28–30). Therefore, measurement should be procured within the confines of the renal cortex in transverse plane. If the pelvis is located intrarenal, the maximum calyx diameter measurement becomes important in addition to the measurement of APRPD in patients with hydronephrosis. According to a recent study combining the presence of diffuse calyceal dilatation with standard APRPD grading, the first postnatal US provides more information for clinical management and improves the predictive probability of surgery (31). It is also reported in another study that pelvic dilatation with calyceal dilatation may be associated with worse postnatal outcomes than pelvic dilatation without calyceal dilation (32).

APRPD measurement has also a predictive importance in determining whether renal function loss occurs. Previous studies in neonates revealed that an APRPD of >6 mm implies obstruction, while a diameter >15 mm is highly accurate in distinguishing infants with severe uropathy (sensitivity and specificity,>90%) (33–36).

Dias et al. reported that combination of prenatal and postnatal APRPD, with cutoffs of 16 and 18 mm, respectively, was 100% sensitive and 86% specific for predicting surgical intervention for UPJ obstruction (33). Burgu et al. found that an APRPD of <20 mm correlated with the persistence of differential renal function. Stable or decreased APRPD on serial US examinations has predictive value to retained or improved function, postnatally (36). In Coplen's study, 15 mm threshold was used, with a 73% sensitivity and 82% specificity for predicting urological obstruction (37). In Sharifian et al.'s study the best APRPD cutoff to predict surgery was 15 mm (38). Dhillon et al. concluded that in the setting of preserved differential renal function (>40%), all patients in their study (*n* = 36) had APRPD of ≥40 mm and experienced renal deterioration requiring surgical intervention while no patients with renal pelvic diameters of <15 mm progressed to surgery (30).

## Society for Fetal Urology (SFU) Grading System

The SFU classification system was developed to replace the traditional grading system, which uses the subjective descriptors “mild,” “moderate,” and “severe.” The SFU grading system is the most widely used grading system in assessment of hydronephrosis in the postnatal period (27).

The SFU grading system is a qualitative assessment of hydronephrosis in determining the degree of dilatation which describes the degree of hydronephrosis according to renal pelvic dilatation, calyceal dilatation, and the presence of cortical thinning. It is classified as grade 0 = no hydronephrosis, grade 1 = only visualized renal pelvis, grade 2 = dilatation of a few but not all calyces, grade 3 = dilatation of virtually all calyces, and grade 4 = dilatation of the renal pelvis and calyces in addition to parenchymal thinning (19). According to the SFU system the status of the calices is more important than the size of renal pelvis. Although the SFU is a useful system, it can be influenced by the practitioner.

Some studies have demonstrated that the severity of hydronephrosis in the SFU grading system correlate with postnatal outcomes. Hydronephrosis with high SFU grades exhibit various features that result in a less predictable prognosis, whereas hydronephrosis with low SFU grades show good prognosis and resolve spontaneously (39). For example, Ross et al.'s study examined neonatally diagnosed patients with grade 3 or 4 hydronephrosis, who were followed up with serial diuretic renography. The study deduced that patients with grade 4 hydronephrosis were more susceptible to having impaired renal function or decreased drainage relative to patients with grade 3 hydronephrosis, making the former more likely to require surgical intervention (40).

SFU grading system has limitations such as being qualitative and subjective; the system is unable to consistently discern diffuse and segmental parenchymal thinning, and the difference between grade 3 and 4 disease remains unclear (41). Similarly, two separate cases that should have different management are defined in the same grade (SFU-4): hydronephrosis with a slightly thinned parenchyma, and a slightly reduced function with hydronephrosis with severely thinned parenchyma and a very severe loss of renal function. To address this shortfall, Sibai et al. (31) suggested the subcategorization of SFU grade 4 as two groups: segmental cortical thinning (grade 4A) and diffuse cortical thinning (grade 4B) (42). In the literature there are also some studies combining the SFU with APRPD (31, 43, 44). Dos Santos et al. proposed a grading system conjoining SFU and APRPD quartiles of <6, 6–9, 9–15, and >15 mm. They additionally included the presence of diffuse caliectasis as a factor in grading (31). In an another study, Longpre et al. offered that grade 4 hydronephrosis and a starting APRPD >29 mm holds predictive value for surgical intervention (44).

## UTD Classification

Established in 2014, the Urinary tract dilation (UTD) classification system is a system developed by representatives from societies which specialize in the diagnosing and treatment of fetuses and children with hydronephrosis. The corresponding eight societies comprise the following: American College of Radiology, American Society of Pediatric Nephrology, Society for Fetal Urology, American Institute of Ultrasound in Medicine, Society for Maternal-Fetal Medicine, Society for Pediatric Radiology, Society for Pediatric Urology, and Society of Radiologists in Ultrasound (20).

The UTD classification system describes the urinary system with the use of six US findings: (1) APRPD, (2) calyceal dilation with distinction between central and peripheral calyces postnatally (central calyces in place of major calyces and peripheral calyces in place of minor calyces), (3) thickness of renal parenchyma, (4) appearance of renal parenchymal, (5) bladder abnormalities, and (6) ureteral abnormalities (20).

While there are only three antenatal subclassifications (normal, UTD A1, UTD A2–3), four subclassifications are defined in the postnatal period (normal, low risk (UTD P1); intermediate risk (UTD P2); and high-risk (UTD P3) (45).

The criteria of the postnatal classification are made regardless of the child's age. According to this classification system a normal kidney has an APRPD of <10 mm and should have no calyceal or ureteral dilation. If the APRPD measurement is between 10 and 15 mm or has central calyceal dilation, the urinary tract is classified as UTD P1. If the APRPD is >15 mm or peripheral calyces are dilated, it is classified as UTD P2. Classification is based on the most concerning US finding, if there is ureteral dilation with APRPD >10 mm it is evaluated as UTD P2. Accompanying with urinary tract dilation of either the renal parenchymal echogenicity, thickness or bladder is abnormal, it is upgraded to UTD P3 (45).

This classification system can be used in prenatal and postnatal evaluation with some advantages over SFU, since it also provides information about ureter and bladder. However, if the cause of hydronephrosis is only due to UPJ obstruction it is not advantageous to include these two parameters, and mentions of superiority would be unsubstantial. Its complicated nature is also a disadvantage for routine clinical practice.

## ONEN Classification

In 2006, Onen proposed an alternative grading system by modifying the SFU grading system to display better the severity of dilatation and to enable easier follow-up in the prenatal and postnatal period evaluations. The system maintains that APRPD is affected by various factors and parenchymal thickness is a more important criterion and relies on the appearance of hydronephrotic kidney, the thickness of renal parenchyma, and the presence of caliceal dilatation. Regardless of the APDRP, severity of hydronephrosis is defined by the degree of caliceal dilatation and of renal parenchymal loss. Grade 1 represents pelvic dilatation alone, Grade 2 with calyceal dilation, Grade 3 with <50% loss of the renal parenchyma, and Grade 4 with severe loss of renal parenchyma (21). While the Onen grade 1 is a combination of SFU grades 1 and 2, SFU grade 4 is divided into two grades (<50% renal parenchymal loss as Onen grade 3; more than 50% renal parenchymal loss as Onen grade 4) (21, 46). The system has been upgraded. Findings such as the absence of corticomedullary differentiation, cortical parenchyma <3 mm, the loss of medullary parenchyma, and significant hyperechogenicity have also been defined AGS grade 4 (47). In our opinion these parenchymal details contribute significantly in the assessment of UPJ obstruction cases.

In addition to these classification systems, alternative several sonographic parameters have been proposed to assess the severity of the hydronephrosis such as pelvicalyceal area (48), parenchymal to pelvicalyceal area (hydronephrosis index) (49), calyx to parenchymal ratio (50), and pelvicalyceal volume using three dimensional (3D) US (51). These methods are more complicated to perform, neccessitate specialized software, therefore they are not commonly utilized in routine clinical practice.

**Figure 5:**
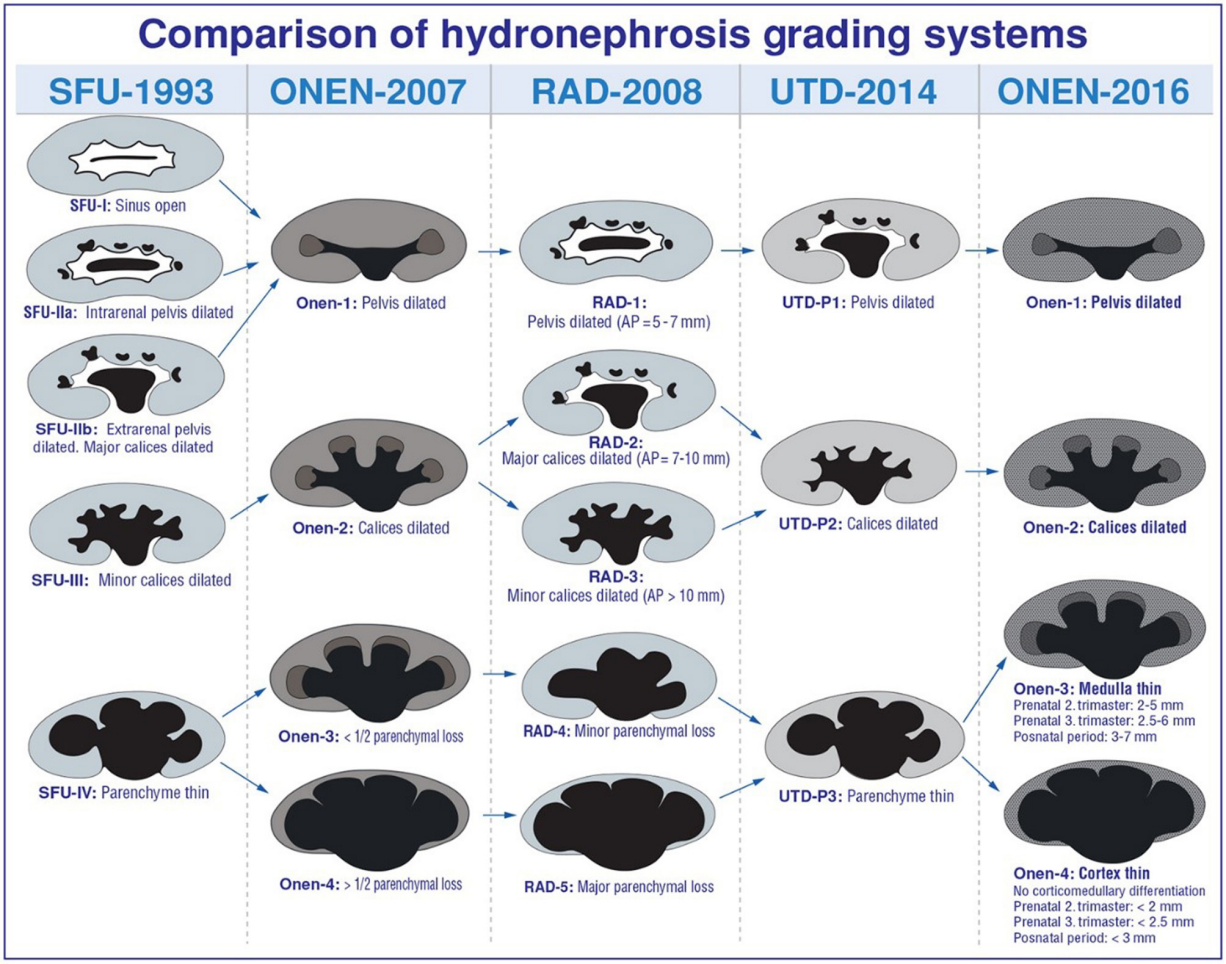
Comparison of hydronephrosis garding system.

**Figure 6:**
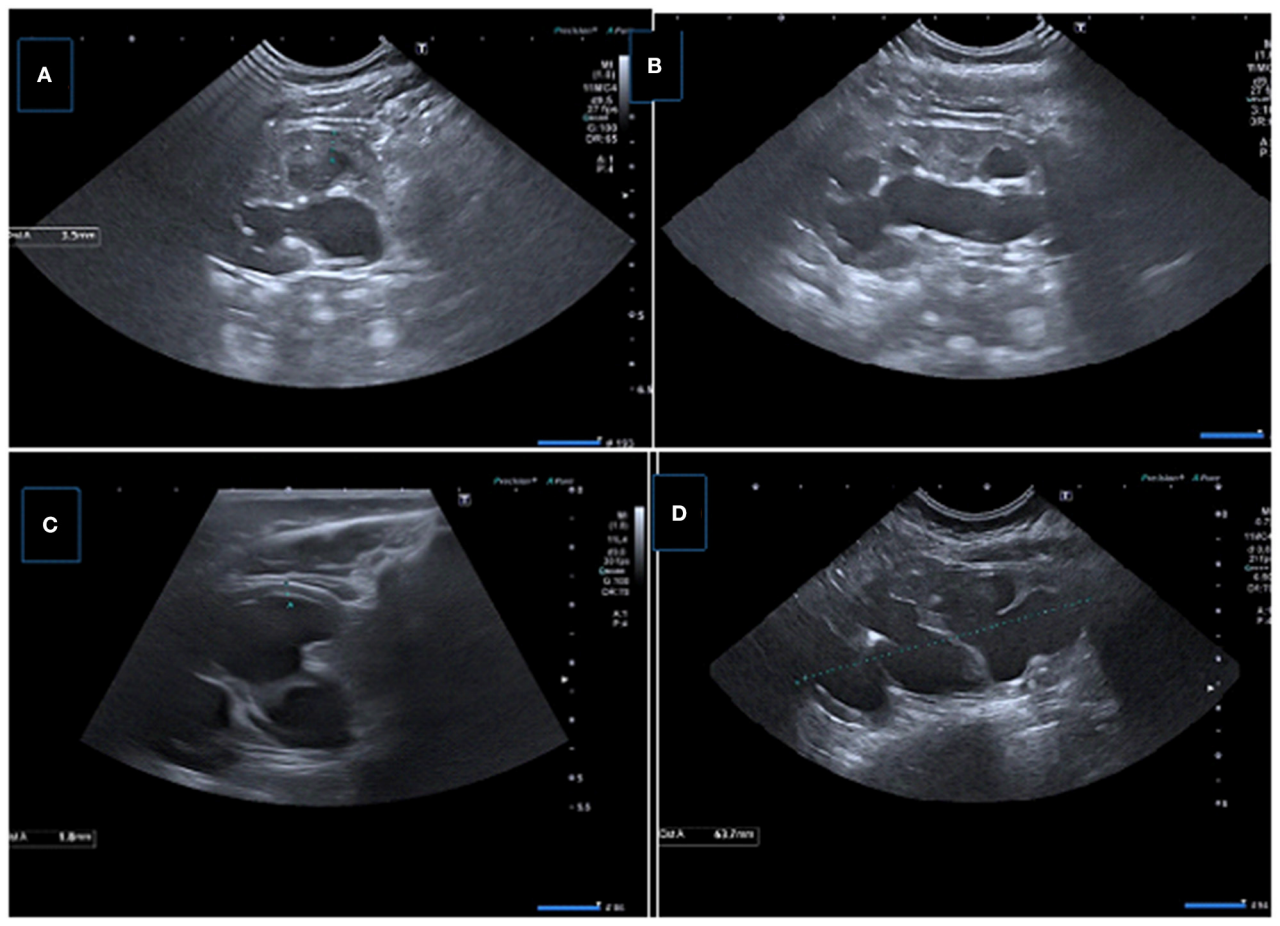
Two consecutive US examinations in a 6-month-old girl with UPJ obstruction. **(A,B)** Baseline US images demonstrate decreased parenchymal thickness with pelvicalyceal dilatation. **(C,D)** Control (2 months later) US images showing significant decrease in renal parenchymal thickness with worsening pelvicalyceal dilatation. A new small echogenic focus suggesting microlithiasis is also present.

**Figure 7:**
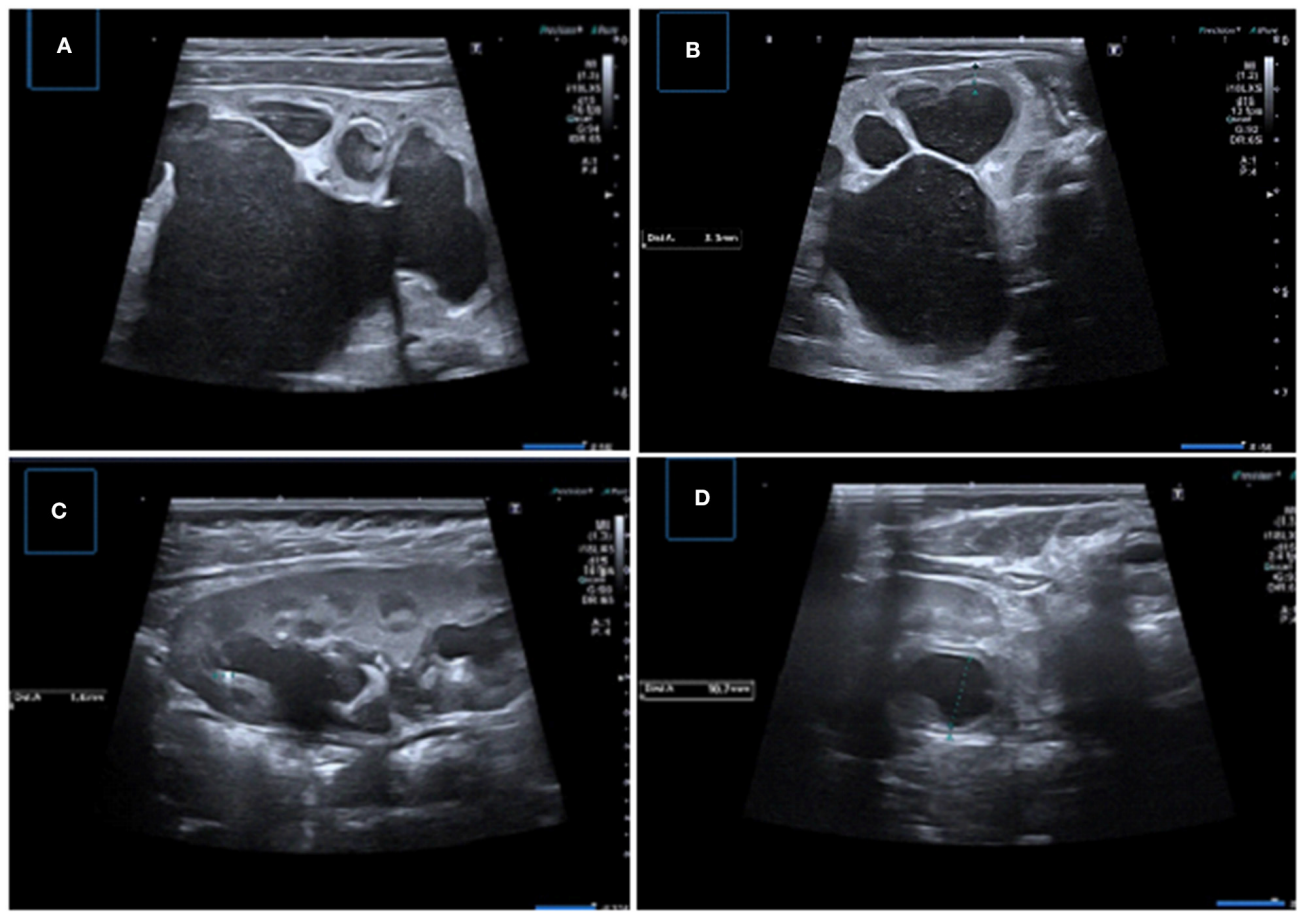
Samples of important US findings in giving surgical desicion. **(A,B)** Longitudinal and transverse US images of the left kidney demonstrating severe pelvicaliyceal dilatation with serious parenchymal thinning, medullary compression, and echogeneity and luminal debris suggesting infection and/or cristalury. **(C)** Longitudinal US image showing pelvicalyceal dilatation with the presence of micro calculi, **(D)** Transverse US image showing uroepithelial thickening and layering of low-level debris consistent with pyonephrosis.

In the literature, many studies comparing these classification systems reported different results with some superiorities and predictive values for surgery (46, 52, 53). There is no definitive standardized imaging algorithm, classification systems, or consensus in terms of necessity of surgical intervention and follow-up (Figure 5). As a result, the current approach is mostly based on a physician's or institutional individual's practice. The decision for surgery is determined based mainly on the severity of hydronephrosis on US, impairment of kidney function in renal scintigraphy, unilaterality, or bilaterality of hydronephrosis and the presence of clinical symptoms including pain, infection, and renal stones (5, 21, 28, 53, 54). US is used as a primary diagnostic tool during follow up of hydronephrosis (7, 13, 17, 55). It is very important to accurately determine whether there is an increase in hydronephrosis on US. Hafez et al. showed the importance of US examination in the follow up of hydronephrosis patients (55). Worsening of hydronephrosis on two successive US scans is considered an indication for the necessity of surgery as it suggests deterioration in renal functions (Figure 6) (54–56). In addition to worsening of hydronephrosis on follow-up US, it is very important to identify the findings that may develop secondary to urinary stasis such as infection or stone development (Figure 7). As management decisions are made based upon consecutive examinations, we suggest US scans be performed by the same practitioners with the same US device, under standardized circumstances and protocols.

In our institution according to the age and consciousness of the child we perform US examination with the bladder full and then emptied. By means of urinary US, drawing from the previous classification systems mentioned above, instead of using classification systems we report all the US measurements and findings of the patient's urinary tract such as; renal size (craniocaudal and axial), location of pelvis (intrarenal or extrarenal), APRPD, calyceal dilatation (central or peripheral), parenchymal thickness, the condition of the renal parenchyma (echogenicity of cortex and medulla, medullary compression, existence of cyst), ureter (caliber, peristalsis, lateralization of the ureterovesical junction, ureteric jet), bladder (capasity, luminal echogenicity, and wall thickness), status of constipation, and possible accompanying urinary malformations. In a pediatric nephrourology council consisting of pediatric nephrologists, pediatric urosurgeons, pediatric radiologists, and nuclear medicine specialists, we discuss the children with all the data collected from the radiologic (prenatal, postnatal, and follow-up), and scintigraphic examinations, paying special attention to the patient's clinical status. A decision is then made for either surgical intervention or follow-up.

## Doppler Ultrasonography

Color doppler US may identify a crossing vessel, when present. The UPJ obstruction due to crossing vessel is one of the extrinsic causes of obstruction that occurs at higher ages than intrinsic causes (3). These vessels usually supply the lower pole of the kidney and most of the time originate from the renal artery or the aorta. Since its treatment is surgical, it is important to detect the presence of a crossing vessel.

Color doppler US might also allow to differentiate a dilated pelvicalyceal system from prominent vessels in the hilum of kidney. Furthermore, assessment of ureteric jets in the bladder can be used to differentiate obstructive causes of hydronephrosis from non-obstructive ones in children. In the presence of obstructive hydronephrosis, the frequency of ureteric jets on the affected side may be greatly reduced when compare with the contralateral normal side (57, 58).

Traditional US does not provide functional data about obstruction. With the use of pulsed doppler, obstructive hydroneprosis can be distinguished from non-obstructive hydronephrosis by renal arterial resistive index (RI) measurements (59, 60). RI is described as the peak systolic velocity minus the lowest diastolic velocity divided by the peak systolic velocity. Because of vasoconstriction caused by renin, angiotensin, and other hormones, diastolic arterial flow velocities are decreased and RI values are elevated in patients with obstructive hydronephrosis (61). A RI of >0.7 and a RI difference of >0.08 between kidneys in children are suggestive of renal obstruction, while a RI of <0.70 generally indicates non-obstructive dilation (59). An elevated RI is not a characteristic finding for obstruction, the value could be >0.70 without obstruction, in patients with renal parenchymal diseases. It should also be remembered that RI values may be higher than that of adults during the newborn and infant period (0.70–1.0). Furthermore, hypotension, a low heart rate, and dehydration can alter the RI values. Nevertheless, a normal RI values are still an important parameter in order to exclude obstruction (62).

## Elastography

US shear-wave elastography (SWE) with acoustic radiation force impulse technology, is a non-invasive, non-ionizing imaging method that might be used to evaluate the stiffness of tissues. In the presence of UPJ obstruction, back pressure from upper urinary tract obstruction may affect renal parenchymal stiffness. A preclinical animal model investigation by Gennisson et al. (63) reported a progressive linear increase in renal stiffness related to increasing urinary pressure. Sohn et al. (64) found that SWE values were higher in kidneys with high-grade hydronephrosis than in normal kidney. In another study by Habibi et al. (65) showed different results: SWE values were higher in control kidneys compared with kidneys affected by UPJ obstruction. In Dillmann et al.'s study to distinguish obstructive hydronephrosis from non-obstructive ones was found no difference in SWE between two groups (66). In addition to limited experience with SWE technology to evaluate kidney, it is not a practical imaging method in the assessment of younger children and requires special application.

## Abdominal Radiographs

Abdominal radiographs may show soft tissue fullness, bulging of the flank from the affected side and status of bowel loops (i.e., constipation). It may also demonstrate possible stone formation in the effected kidney and give information about the lumbosacral vertebraes.

## Voiding/Micturating Cystourethrogram

As this imaging modality will be discussed in detail within the scope of this journal as a separate article, we want to mention only briefly.

The voiding/micturating cystourethrogram cannot evaluate the obstruction but enables to exclude other causes of hydronephrosis, including accompanying vesicoureteral reflux (VUR), urethral valves, and ureteroceles (67). VUR may coexist with UPJ obstruction in 8–14% of cases. Identification of VUR is important since children with concurrent VUR and UPJ obstruction may have increased risk for infection (68). Because of its invasive nature, radiation exposure, the risk of urinary tract infection after procedure, indications of voiding cystourethrography should be carefully determined. In the presence of bilateral hydronephrosis (or solitary kidney), duplicated system, small kidney, abnormal echogenicity, dilated ureter, ureterocele, suspected infravesical obstruction, and abnormal bladder voiding cystourethrogram should be performed (69).

## Intravenous Pyelography

Intravenous pyelography (IVP) or intravenous urography (IVU) has been the important imaging modality for assessment of the urinary tract (70). Although IVP indications have decreased with advances in imaging technology, it is still used in some centers where advanced imaging methods are limited. Dilatation of collecting system, with parenchymal changes in the nephrogram phase, and delay in excretion of contrast medium are characteristic findings of obstructive hydronephrosis (71). But IVP is not sufficient for visualization of poorly functioning kidneys which are severely blocked due to poor contrast excretion (72). It has some disadvantageous such as impaired image quality as a result of bowel gas, the risk of radiation exposure, contrast nephrotoxicity, and hypersensitivity reactions. It may also requires several radiographs with total examination time period extending up to many hours in cases of urinary tract obstruction.

## Computerized Tomography Urography and Angiography

In spite of all advances, as a rule, computed tomography (CT) must be avoided in pediatric patients because of the x-ray content as much as possible (73). Despite ionizing radiation exposure, it can be useful in some specific indications in kidneys and urinary tract diseases in children (74). This method should be considered as a second line imaging technique in children; it can support the diagnosis after a comprehensive US evaluation including Doppler US. CT scan can detect the location and cause of obstruction such as crossing vessels

While maintaining the diagnostic value of CT examinations as in the ALARA principle, it should be aimed to minimize the dose of X-ray radiation as in the ALARA principle (75–77). For this purpose, the patient should be evaluated with age-adapted kVp and mAs values, multi-phase examinations should be avoided and appropriate amount of contrast, and delay time should be selected (77). If IV contrast medium administration injection is necessary, low or iso-osmolar and non-ionic iodinated ones should be administered and renal function must be checked prior to the examination. Children should be hydrated before the examination. Contrast agent dose may range from 1 and 4 ml/kg, generally 2 ml/kg (78). Since the scan times is shorter, sedation is not often needed.

Multidetector CT scanners allow for rapid and complete imaging of the urinary tract and comprehensive evaluation of the urinary system pathologies. Thin CT slices thickness of <1 mm provides optimal reconstruction in coronal and sagittal planes. The sagittal-coronal projections, additional 2D and 3D-reconstructions 3D-volume rendering and maximal intensity projection (MIP) images are very helpful in better visualizing the anatomy of the collecting system and as well the crossing vessel. Application of CT in the assessment of the urinary tract is called CT urography (CTU), vascular structures evaluation is called CT angiography (CTA).

CTU examination is used for imaging the kidneys and urinary tracts, where the excretory phase is mandatory (79). The triple-phase technique includes separate non-enhanced, contrast- enhanced, and excretory phases. Non-enhanced phase may be obtained to detect stones that may occur secondary to obstruction. On contrast enhanced excretory phased CT, the obstructed kidney demonstrates delayed opacifications, and excretions of contrast material. But it is essential to remember the increased radiation exposure risk of multi-phase studies in children. Therefore, several imaging protocols have been used in practice, in order to decrease radiation exposure such as split bolus technique (80). The contrast medium is administered in two parts, with a several minutes interval between the portions. A split bolus of the contrast agent, combining the parenchymal, and excretory phases may help to reduce the need for multiple phases in some conditions. In addition to ensuring that two examination phases during one scan, this protocol reduces the radiation dose while maintaining the diagnostic value of both phases (78, 80).

**Figure 8:**
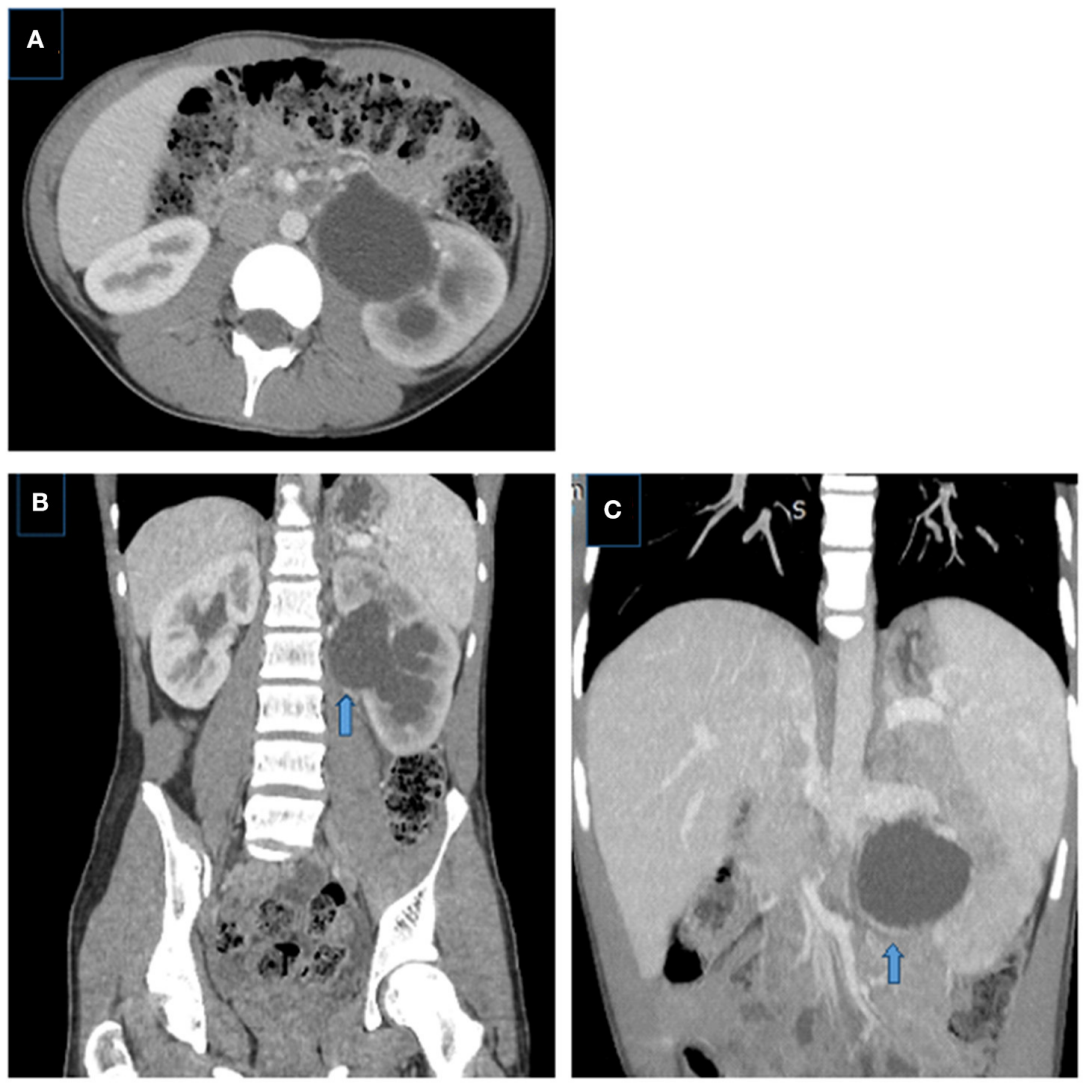
Crossing aberrant renal artery causing left UPJ obstruction in a 14 year-old boy. **(A)** Axial and **(B)** coronal CT images showing left pelvicalyceal system dilatation with delayed nephrogram phase, pelvis is extrarenally located, dilatation is more prominent in the pelvis than calices, note the crossing vessel (arrow). **(C)** Coronal MIP image better demonstrates the crossing vessel as the cause of UPJ obstruction (aberrant lower pole artery) (arrows).

The arterial phase is very important and crucial in order to detect the crossing vessel and CTA with multiplanar reformatted and three-dimensional images are used to evaluate the cause of the crossing vessel as a cause of UPJ obstruction especially in older children (Figure 8) (81).

Although the radiation risk is well-known in pediatric patients, CTU, and CTA examinations provide important information both for anatomy and function of the urinary tract (renal parenchyma, collecting system, accessory vessel, stone formation, and contrast excretion) with higher acquisition speed especially in patients who are unable to undergo MRI or in center where MRI is not available.

## Magnetic Resonance Urography

In recent years, Magnetic Resonance Urography (MRU) has substantially progressed due to the development of high-resolution image generating software and hardware. This imaging technique currently permits the detailed evaluation of complex renal and urinary tract anatomy, while also providing information regarding renal function, including differential renal function, and the presence or absence of obstructive uropathy without the use of ionizing radiation (82, 83). MRU has all the disadvantages of MRI, such as requiring sedation to prevent motion artifacts in younger children. The use of gadolinium, which may be the cause of nephrogenic systemic fibrosis in patients with low glomerular filtration rate (GFR), presence of a metallic prosthesis, staying 35–70 min in an enclosed area for claustrophobic patients and costs are other additional disadvantages (74).

MRU is a promising alternative method, being a single examination able to assess kidneys and the entire urinary tract as it combines both anatomic and functional information (84–86). In addition to providing detailed anatomical and morphological information about the kidney, MRU enables the evaluation of the whole ureter course and identification of ectopic insertions and potential causes of obstruction (such as crossing vessel) (87, 88).

It is possible that a pediatric MRU be performed at 1.5 or 3 Tesla (T) in children of any age by using multi-element phased-array surface coils. 3 T magnets provide better image resolution, whereas 1.5 T magnets tend to provide more homogeneous fat saturation and are less susceptible to artifacts. A bladder catheter is placed, which permits for continual drainage of urine to avert patient discomfort and promote excretion and assessment of the urethra on imaging. The bladder catheter is first clamped to allow evaluation of the bladder, then the catheter is left to drain. A peripheral IV catheter is positioned to administer hydration, diuretic (usually furosemide) and IV contrast material (86).

MRU examination consists of two basic approaches. The first technique allows evaluation of the anatomical structures of the kidney, ureter, and bladder by using a diversity of T2-weighted pulse sequences (e.g., single shot fast spin echo, two-dimensional fast spin echo [2D] [FSE], and three-dimensional [3D] FSE) (74, 86). It enables direct visualization of UPJ anatomic structures, assessing the degree of luminal narrowing, and determining the presence of UPJ kinking or tortuosity as well as the site of ureteral insertion on the renal pelvis (e.g., abnormally high insertion) (87, 88).

**Figure 9:**
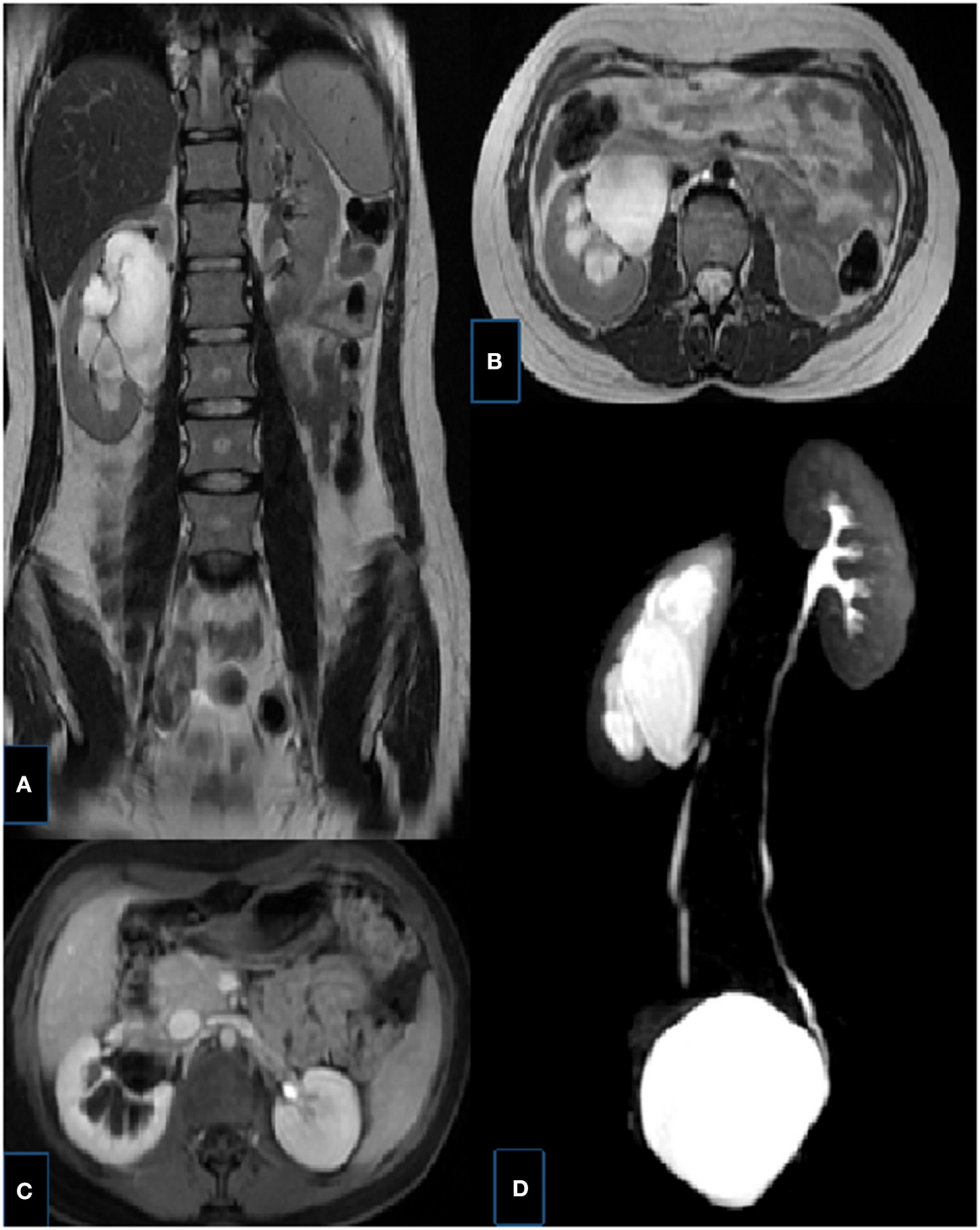
Right UPJ obstruction in a 14-year-old girl. **(A,B)** T2-weighted fast spin-echo coronal (a) and axial (b) MR images showing right renal collecting system dilatation, pelvis is extrarenally located, the thickness of renal parenchyma is decreased and corticomedullary differentiaton is lost. **(C)** Axial post-contrast excretory phase showing delayed excretion in the right renal collecting system, notice the contrast material in the left pelvis. **(D)** MIP MR image showing UPJ obstruction with kinking and angulation at the UPJ and a normal caliber ureter, left kidney is normal.

The second technique involves dynamic and delayed postcontrast MRU images that allow evaluation of renal perfusion (including imaging of renal arteries, quality of parenchymal enhancement, contrast material excretion into the renal collection systems, and ureters). Delayed postcontrast images can also be utilized in generating 2D reformations that provide optimal visualization of relevant anatomic structures (e.g., the UPJ) and 3D reconstructions, including MIP and volume-rendered images, which provide an overview of urinary tract anatomic structures on a single image (Figure 9). This method also allows the measurement of differential renal function [based on the amount (volume) of enhancement of renal parenchyma or based upon glomerular filtration of contrast material] and time vs. signal intensity washout/excretion curves. Currently, accurate absolute quantification of glomerular filtration rate is not possible with MRU (89).

MRU is a promising imaging modality with superior anatomical and functional information in a single test free of the use of ionizing radiation and functional MRU might be able in the future to replace the renogram, because of the quality of the signal. However, due to difficulties of implementation in pediatric group, the absence of each center and the need to increase experience in this regard, it is not widely use yet.

## Post-Operative Evaluation

Many modalities have been used, US, IVP, radionucleotide scan (RS), and MRU to evaluate patients in postoperative period at various time intervals. US and RS are the most widely used investigations (90). As in pre-operative evaluation of UPJ obstruction, there is also no consensus about the follow-up approach and interval in the post-operative period. Studies suggest that follow-up can be performed with both US and RS at certain time intervals in the postoperative period which can direct the necessity of further investigations (91, 92). However, it is obvious that the US should be the first choice to avoid both radiation and urethral catheterization with an increased risk of urethral trauma and urinary system infections in pediatric patients. If there is suspicion about complications in post-operative periods such as urinary tract infections, pyelonephritis, urine extravasation, US is also the first imaging modality.

**Figure 10:**
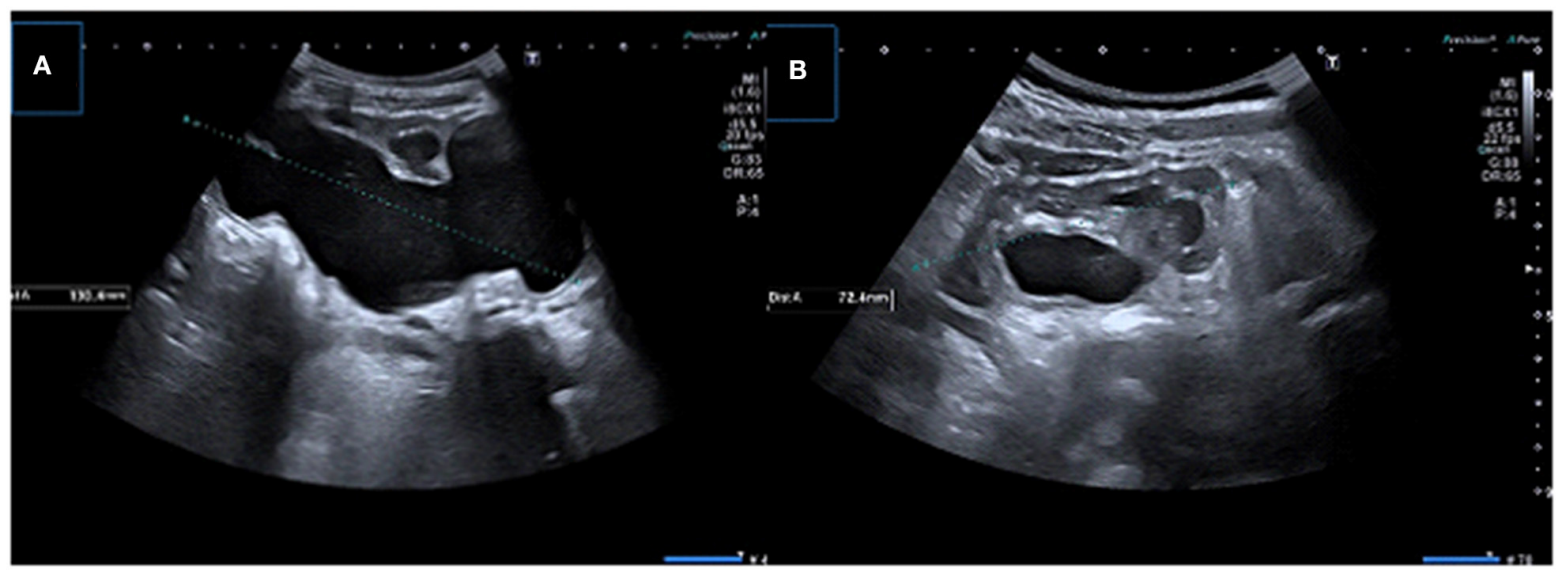
**(A)** Longitudinal US image in a 1-month-old boy infant showing significant dilation of the pelvicalyceal system with parenchymal thinning **(B)** Control US image obtained after pyeloplasty demonstrates significant resolution of dilatation.

Properly performed US provides an accurate assessment of renal pelvis/caliceal dilatation, renal parenchymal thickness, echogenicity, and renal growth postoperatively. After successful pyeloplasty, renal function stabilization takes ~1 year and renal function may improve (Figure 10). If there is no problem in the early postoperative period, first control with urinary US may be performed 1 month after the operation. Persistance of the pelvicalyseal dilatation does not indicate continued obstruction (93). In this early post-operative period, significant resolution of hydronephrosis should not be expected, no worsening, or a slight decrease in hydronephrosis can be sufficient (93). Because even if obstruction is surgically removed, the average time for the renal pelvis to regain flexibility is achieved around 2 years (21, 30). On the other hand, it should also be known that early improvement in dilatation on US could be due to surgical reduction of the renal pelvis rather than true improvement. Measurement of pelvis AP diameter and parenchymal thickness may be useful for follow-up but there is no cut-off value in pelvic diameter due to these factors mentioned above and the level of hydronephrosis is also affected by hydration or the amount of urine in the bladder. However, we can say that worsening or persistence of hydronephrosis, decrease in cortical thickness and clinical findings (i.e., colic pain, urinary tract infection) are not expected findings and should alert to determine the functional patency of the UPJ.

Although majority of surgical failures occur within 1 year after pyeloplasty, there are also cases reported later and failure rate has been described in published reports as 5–10% (94, 95). Serial renal US are recommended at 3, 6, and 12 months, and then annually for 2 years, with additional testing based on US and clinical presentation (95).

IVP was previously widely used to assess surgical success after pyeloplasty, although it is not preferred now. CT and MRU are other radiological options to assess surgical anastomoses (e.g., in the context of UPJ obstruction repair) and reimplanted ureters (96).

## Conclusion

US is the main imaging study used to diagnose UPJ obstruction. This method has lots of advantages but does not provide functional information about the urinary tract. The question is to differentiate true obstruction from urinary tract dilatation which is very crucial in determining the treatment decision. US examination provides essential information regarding laterality, kidney size, appearance (such as echogenicity, corticomedullary differentiation, cyst), parenchymal thickness, degree of obstruction. In order to provide right decision, necessity of surgery and standardization, grading, and classification systems have been developed. However, there is no definite consensus and worldwide accepted standard protocols and as a result current therapeutic approach is mostly based on US findings, follow-up results, clinical and scintigraphic findings, and dependent on physician or institutional individual practices. CT and MR are not routinely performed radiologic studies but are often reserved for special cases such as demonstration of an aberrant artery as the cause of obstruction.

## Author Contributions

All authors listed have made a substantial, direct and intellectual contribution to the work, and approved it for publication.

## Conflict of Interest

The authors declare that the research was conducted in the absence of any commercial or financial relationships that could be construed as a potential conflict of interest.
